# Preparation of Mouse Monoclonal Antibody for RB1CC1 and Its Clinical Application

**DOI:** 10.1371/journal.pone.0032052

**Published:** 2012-03-01

**Authors:** Yusuke Hama, Tokuhiro Chano, Takuma Inui, Kyoichi Matsumoto, Hidetoshi Okabe

**Affiliations:** 1 Department of Clinical Laboratory Medicine, Shiga University of Medical Science, Shiga, Japan; 2 Mikuri Immunology Laboratory, Osaka, Japan; Bauer Research Foundation, United States of America

## Abstract

RB1-inducible coiled-coil 1 (RB1CC1; also known as FIP200) plays important roles in several biological pathways such as cell proliferation and autophagy. Evaluation of RB1CC1 expression can provide useful clinical information on various cancers and neurodegenerative diseases. In order to realize the clinical applications, it is necessary to establish a stable supply of antibody and reproducible procedures for the laboratory examinations. In the present study, we have generated mouse monoclonal antibodies for RB1CC1, and four kinds of antibodies (N1-8, N1-216, N3-2, and N3-42) were found to be optimal for clinical applications such as ELISA and immunoblots and work as well as the pre-existing polyclonal antibodies. N1-8 monoclonal antibody provided the best recognition of RB1CC1 in the clinico-pathological examination of formalin-fixed paraffin-embedded tissues. These monoclonal antibodies will help to generate new opportunities in scientific examinations in biology and clinical medicine.

## Introduction

RB1-inducible coiled-coil 1 (RB1CC1: the symbol used here, which is approved by the Human Genome Organization [HUGO] Gene Nomenclature Committee; it is also known as FIP200, focal adhesion kinase family-interacting protein of 200 kDa) plays an important role through its interaction with various molecules in several cell signaling pathways. RB1CC1 is a positive regulator of the NF-kβ signaling pathway [1], [2] and mTOR [1], [3], [4]. RB1CC1 also functions as a negative factor in FAK-Erk1/2 signaling [5], [6]. Importantly, RB1CC1 has been identified recently as a mammalian homolog of Atg17 in the autophagy pathway [7], [8], [9], [10], [11].

In mammalian cell nuclei, RB1CC1 forms a complex with p53 and/or hSNF5, and the large protein complex works as a strong activator of *RB1*, *p16*, and *p21* promoters [12], [13], [14], [15], [16]. Thus, nuclear RB1CC1 enhances the RB1 pathway globally and behaves as a tumor suppressor in some types of human malignancies [17], [18].

RB1CC1 has been established as a prognostic predictor in breast and salivary cancer patients [17], [18]. Thus, a nuclear RB1CC1 expression has been revealed to be fundamentally related to better prognoses in breast and salivary cancers. Furthermore, the combined evaluation of RB1CC1, RB1, and p53 can significantly predict a longer disease-specific survival [17], [18]. Therefore, evaluation of RB1CC1 expression combined with RB1 and p53 status would provide useful information in clinical practice and help the planning of future therapeutic strategies in various human cancers.

In order to make the clinical evaluation of RB1CC1 generally available and to clarify more precisely its biological function, specific antibodies must react reproducibly in various laboratory experiments and be available as a stable supply to clinics and scientific laboratories. Here, we describe the production of mouse monoclonal antibodies that function as well as the preexisting polyclonal antibodies [3], [10], [12], [17], [18], [19], [20] in various experimental and clinical applications; and one of these monoclonal antibodies appears to be particularly suitable for the clinical pathological evaluation of formalin-fixed paraffin-embedded tissue sections.

## Results and Discussion

**Figure 1:**
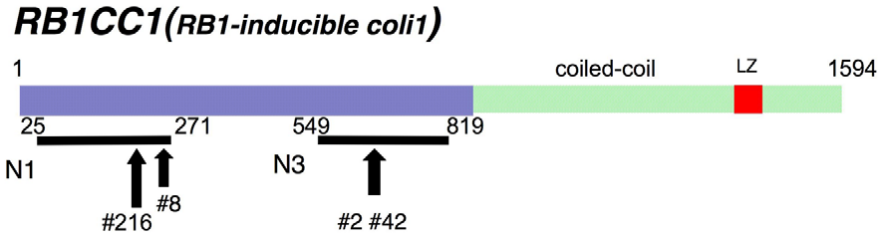
A schematic representation of RB1CC1, immunogens, and epitope sites for four kinds of monoclonal antibodies (N1-8, N1-216, N3-2, and N3-42). Two recombinant polypeptides of RB1CC1 containing amino acid (aa) residues 25–271 (N1) and 549–819 (N3), respectively, were used as immunogens. N1-216, N1-8, N3-2, and N3-42 antibodies recognize epitopes within aa residues 235–254, 255–271, 715–734, and 715–734, respectively, of RB1CC1.

**Figure 2:**
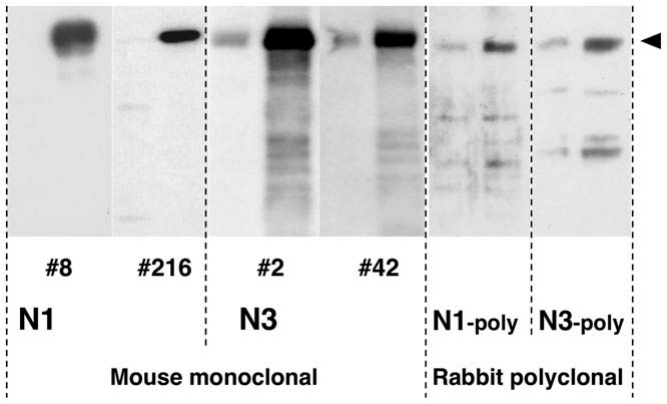
Immunoblot analysis of four monoclonal antibodies and pre-existing polyclonal antibodies. Four kinds of monoclonal antibodies (N1-8, N1-216, N3-2, and N3-42) showed strong immunoreactivity for ∼200 kDa full-length human RB1CC1 and little cross-reactivity with other proteins in Western blot analysis. Two kinds of pre-existing polyclonal antibodies (N1-poly and N3-poly) were used to the blotting control. Four µg of protein lysates of HEK293 and the cells overexpressing RB1CC1 were applied to the analysis. An arrowhead shows the position of ∼200 kDa full-length human RB1CC1.

**Figure 3:**
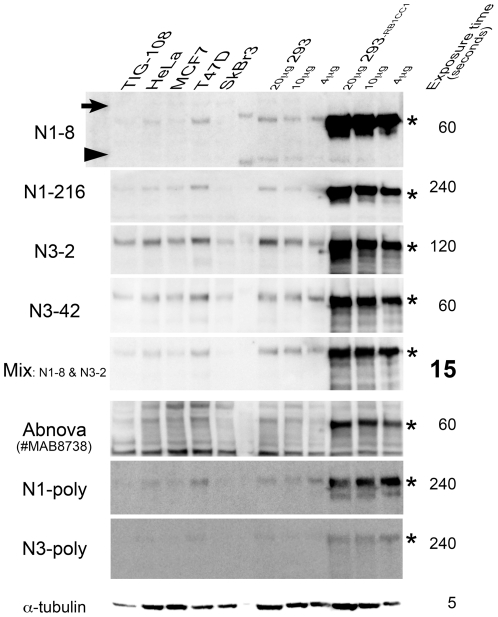
Comparison between monoclonal and polyclonal antibodies for RB1CC1. Western blot analyses using 4 kinds of monoclonal (N1-8, N1-216, N3-2 and N3-42), one commercially available monoclonal (#MAB8738; Abnova), and 2 kinds of polyclonal (N1-poly and N3-poly) antibodies for RB1CC1 are demonstrated in various cell lines. Twenty µg of each cell protein was loaded in each lane of the SDS-PAGE. Twenty, ten and four µg of proteins of HEK293 and the cells overexpressing RB1CC1 were also loaded. α-tubulin was used as a blotting control for protein mounts. The automatically exposure time (ImageQuant™ LAS-4000; Fujifilm, Japan) was indicated at right ends. Arrow, arrowhead and * indicate 250, 150 kDa and full-length RB1CC1, respectively.

In order to generate mouse monoclonal antibodies that recognize RB1CC1, we prepared polypeptides of residues 25–271 (N1) and 549–819 (N3) of RB1CC1 and used them as immunogens (Fig. 1). Each antigen-specific antibody was screened by ELISA, and 112 antibodies for the epitopes within N1 and 58 antibodies for the epitopes within N3 were produced. Among 170 antibodies screened by ELISA, four antibodies were selected by their strong immunoreactivity against ∼200 kDa full-length human RB1CC1 and little cross-reactivity with other proteins in immunoblots. These four antibodies were named N1-8, N1-216, N3-2, and N3-42. In immunoblots, it was confirmed that these four antibodies recognized ∼200 kDa full-length RB1CC1 more specifically and stronger than the previously reported polyclonal antibodies [3], [10], [12], [17], [18], [19], [20] (Fig. 2–3). These antibodies were also more specific than a commercially available one (#MAB8738; Abnova), recognizing some non-specific signals, and were utilized for the signal sensitivities by mixing them (Fig. 3).

**Figure 4:**
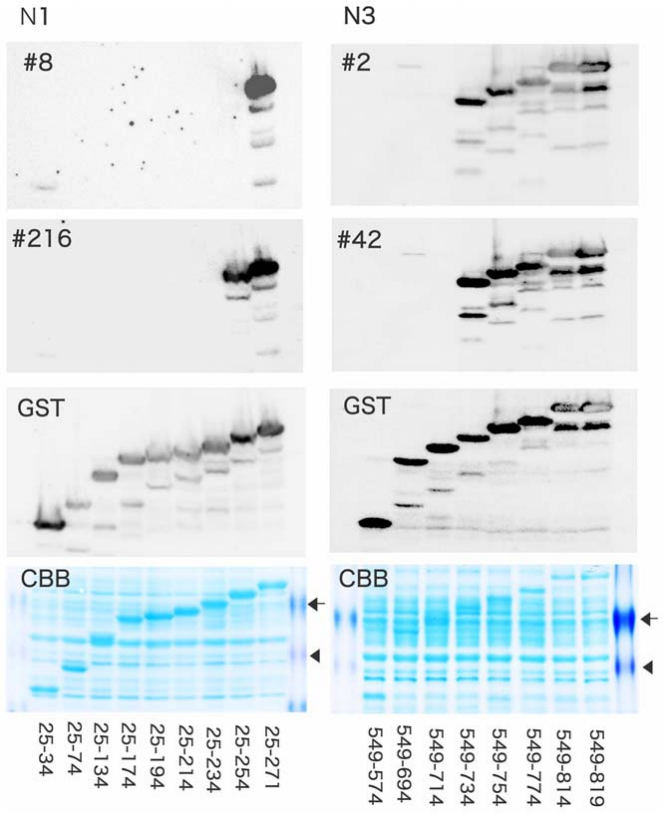
CBB staining and immunoblot analysis of four kinds of monoclonal antibodies (N1-8, N1-216, N3-2, and N3-42). N1-8 did not recognize aa residues 25–254, but recognized aa residues 25–271 of N1. Therefore, N1-8 was considered to recognize an epitope located among aa residues 255–271 of RB1CC1. Similarly, N1-216, N3-2, and N3-42 recognized epitopes located among aa residues 235–254, 715–734, and 715–734, respectively. The recombinant mutant proteins (∼27–56 kDa) of N1 and N3 were confirmed by anti-GST immunoblots. Coomassie Brilliant Blue (CBB) staining of each protein band in polyacrylamide gel electrophoresis is shown in the lowest row. Arrows and arrowheads indicate 47 and 39 kDa, respectively.

To identify precisely each epitope for the monoclonal antibodies, we performed epitope mapping using ∼27–56 kDa smaller mutant peptides generated with stop-codon in N1 or N3 polypeptides. According to the detection of immunoreactivities, N1-8 did not recognize amino acid (aa) residues 25–254, but reacted only with aa residues 25–271 of N1 (Fig. 4). Therefore, N1-8 could recognize an epitope located within the 255–271 aa residues of RB1CC1. Similar strategic experiments suggested that N1-216, N3-2, and N3-42 could recognize epitopes located within the 235–254, 715–734, and 715–734 aa residues, respectively, of RB1CC1.

**Figure 5:**
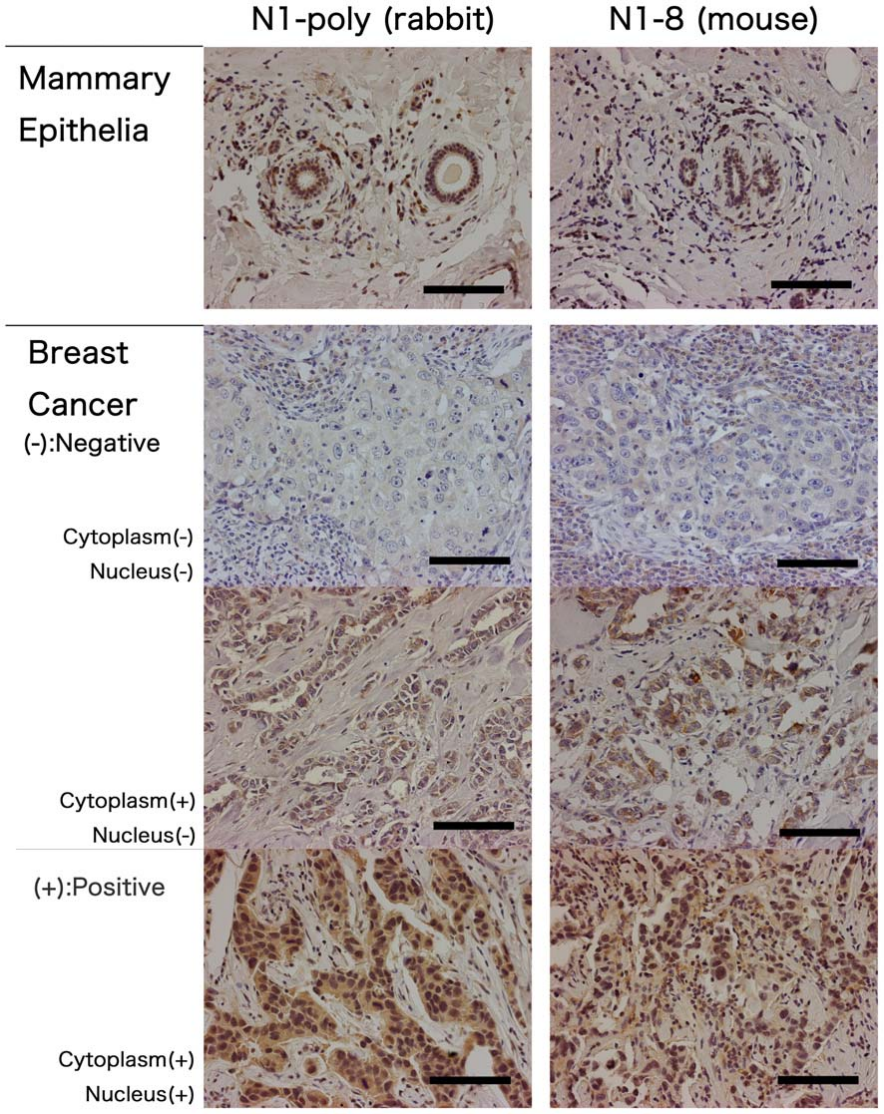
Comparison of immunoreactivities between N1-8 monoclonal and N1 polyclonal anti-RB1CC1 antibodies in formalin-fixed paraffin-embedded breast cancer tissues. In non-neoplastic mammary ductal epithelium, cytoplasm and nuclei were similarly stained with these two antibodies. In breast cancer tissue samples, these two antibodies reacted similarly in all types of staining variation (I, negative staining in both cytoplasm and nuclei; II, positive staining only in cytoplasm; III, positive staining only in nuclei or in nuclei and cytoplasm). I–II and III were defined as RB1CC1 -negative and -positive, respectively, in the previous clinical cohorts. Bar, 100 µm.

In order to find the most useful antibody for clinical pathological applications, we evaluated the immunoreactivities of these four kinds of monoclonal antibodies in formalin-fixed paraffin-embedded tissue sections, comparing the reactivities with that of the pre-existing polyclonal antibody [3], [10], [12], [17], [18], [19], [20]. Among the four monoclonal antibodies, N1-8 had the strongest immunoreactivity in formalin-fixed paraffin-embedded tissue sections, and non-specific reaction was negligible. Therefore, we compared the immunoreactivity of N1-8 monoclonal antibody with that of the rabbit polyclonal antibody [3], [10], [12], [17], [18], [19], [20] using breast cancer tissues. N1-8 monoclonal antibody had a similar immunoreactivity as that of the polyclonal antibody in formalin-fixed paraffin-embedded breast cancer tissue sections containing non-neoplastic mammary ductal epithelium. In non-neoplastic ductal epithelium, both cytoplasm and nuclei were similarly stained with these two antibodies. As reported previously [12], [17], [18], [20], there were three types of staining variations in breast cancer samples: I, negative staining in both cytoplasm and nuclei; II, positive staining only in cytoplasm; III, positive staining only in nuclei or in nuclei and cytoplasm. N1-8 monoclonal and the polyclonal antibodies reacted similarly to RB1CC1 in the formalin-fixed paraffin-embedded breast cancer tissue samples (Fig. 5).

In a series of 57 clinical cases of breast cancer, immunoreactive variations between N1-8 monoclonal and the pre-existing polyclonal antibodies were precisely evaluated. Although N1-8 could recognize cytoplasmic RB1CC1 better than the polyclonal antibodies, both antibodies reacted identically with nuclear RB1CC1 (Table 1), which can predict a better long-term prognosis of breast and salivary cancer patients [17], [18], [20]. N1-8 appears to be best adapted to the clinical pathological evaluation of RB1CC1 expression in human pathological disorders, such as various cancers and neurodegenerative diseases.

**Table 1:** Summary of immunoreactive variations between N1-8 monoclonal and polyclonal antibodies in a series of 57 clinical breast cancer cases.

			N1-8 monoclonal			
		(−)		(+)	Cases	
		cytoplasm				
		(−)	(+)			
N1-polyclonal						
	cytoplasm					
**(−)**	(−)	4	5	0	9	
	(+)	2	8	0	10	19
**(+)**		0	0	38		38
		6	13			
			19	38		57

Although N1-8 could recognize cytoplasmic RB1CC1 better than the polyclonal antibody (13 vs 10 cases), both antibodies were completely consistent in recognizing the nuclear reactivity of RB1CC1.

In conclusion, we have produced four kinds of monoclonal antibodies that can specifically recognize and quantify RB1CC1 in examinations such as ELISA and immunoblots. One monoclonal antibody (N1-8) had the best ability to recognize RB1CC1 in formalin-fixed paraffin-embedded tissue sections; this antibody can be supplied stably to clinico-pathological laboratories.

## Materials and Methods

### Immunization and preparation of hybridomas

In order to prepare immunogens, two kinds of recombinant partial fragments of RB1CC1 were produced in BL21 E. coli. N1 consisted of aa residues 25–271 and contained the NH2-terminal glutathione-S-transferase (GST); N3 consisted of aa residues 549–819 and similarly contained the NH2-terminal GST. Each of them also contained the COOH-terminal polypeptide fragment of RB1CC1 (GenBank accession No. NP_055596). N1 and N3, whose molecular weights were 54 and 56 kDa, were used as immunogens after purification via SDS-polyacrylamide gel electrophoresis (SDS-PAGE). BALB/C mice were immunized with these purified proteins in complete Freund's adjuvant. Hybridoma clones that produced each antigen-specific antibody were first screened by ELISA and then by Western blot analysis.

### Western blot analysis

HEK 293 cells and cells overexpressing RB1CC1, which had been transiently transduced with pcDNA3.1-RB1CC1 [13], were lysed for Western blotting, as described by Sarbassov et al [21]. After clearing lysed materials by centrifugation at 15,000× g for 10 min, the supernatants were boiled in SDS sample buffer. Proteins resolved by SDS-PAGE were transferred to polyvinylidene difluoride (PVDF) filters. After blocking of the filters with TBS-T (10 mM Tris-HCl (pH 7.6), 150 mM sodium chloride, 0.1% Tween 20) containing 5% bovine serum albumin (BSA), the filters were incubated overnight with the primary antibodies (2–4 µg/ml IgG) in TBS-T containing 2% BSA at 4°C. The filters were then washed in TBS-T and incubated for 1 h in horseradish peroxidase-conjugated anti-mouse IgG diluted 1∶10,000 in TBS-T containing 2% BSA. After several washes with TBS-T, the immunoreactivity was detected using the ECL system (GE Healthcare, UK) according to the procedures recommended by the manufacturer.

Totals of 112 and 58 mouse monoclonal antibodies specifically reacted with N1 and N3 polypeptides, respectively, in ELISA. Four antibodies were additionally selected by immunoreactivity for ∼200 kDa full-length RB1CC1 in immunoblots. These antibodies have been named N1-8, N1-216, N3-2, and N3-42 in the present study, and they are presently available for commercial purchase (Mikuri Immunology Laboratory, Osaka, Japan).

### Comparison between monoclonal and polyclonal antibodies for RB1CC1

The cancer cell lines, HeLa (endocervical carcinoma), MCF7, T47D, and SKBr3 (breast carcinoma) were purchased from American Type Culture Collection. TIG-108 human normal fibroblast was from the Japanese Collection of Research Bioresources. These cell lines were cultured in Dulbecco's modified Eagle's medium or RPMI 1640 containing 10% FBS. All cell culture media were supplemented with penicillin (50 units/mL) and streptomycin (50 mg/mL). These cell lines were incubated at 37°C in a humidified chamber supplemented with 5% CO2. Endogenous RB1CC1 expressions of each cell line were evaluated by Western blotting using 4 kinds of mouse monoclonal antibodies (N1-8, N1-216, N3-2 and N3-42), a commercially available one (#MAB8738; Abnova, Taiwan), and 2 kinds of pre-existing rabbit polyclonal antibodies (N1-poly and N3-poly) [3], [10], [12], [17], [18], [19], [20]. ImageQuant™ LAS-4000 (Fujifilm, Japan) automatically transferred the blotting signals to digital images, referring to the intensities. The automatically exposure time was inversely reflected to the maximum signal of each blot.

Two kinds of anti-RB1CC1 polyclonal antibodies (N1-poly and N3-poly) were generated in our laboratory as described previously [3], [12], [19]. Briefly, 2 kinds of NH_2_-terminal GST-fusion proteins containing aa residues 25–271 (N1) and 549–819 (N3) of RB1CC1 were used as immunogens in rabbits. IgG fractions were purified from rabbit antisera, and applied to the experiments with 1–2 µg/ml as polyclonal antibodies for RB1CC1.

### Epitope mapping

Epitope mapping experiments were performed to detect the antigen epitopes for each of the selected four monoclonal antibodies. We artificially introduced stop codons into the N1 and N3 peptides. Plasmids containing each of the mutated N1 or N3 peptides were then transfected into BL21 E. coli and expressed: ∼27–54 kDa N1 mutant polypeptides comprised residues 25–34, 25–54, 35–74, 25–94, 25–114, 25–134, 25–154, 25–174, 25–194, 25–214, 25–234, 25–254, and 25–274; ∼29–56 kDa N3 mutant polypeptides comprised residues 549–574, 549–594, 549–614, 549–634, 549–654, 549–674, 549–694, 549–714, 549–734, 549–754, 549–774, 549–794, 549–814, and 549–819. The proteins were lysed in 1× Laemmli's SDS sample buffer. Two sets of sample proteins that were translated from these mutants were prepared and resolved by SDS-PAGE. Then, one set of the samples was stained by Coomassie Brilliant Blue (CBB) using Quick-CBB PLUS (Wako; Osaka, Japan), and another set was transferred to a PVDF filter. The filter was immunoblotted with four different monoclonal antibodies (N1-8, N1-216, N3-2, and N3-42) and anti-GST antibody.

### Immunohistochemistry

To evaluate the reactivities of anti-RB1CC1 monoclonal antibodies with human breast cancer tissues, histological specimens from 57 cases were used in this study. The formalin-fixed paraffin-embedded tissue samples were serially sliced into 5-µm sections. Deparaffinized sections were heated by autoclaving (120°C, 1 min) or ImmunoSaver™ (Nissin EM Ltd., Japan; 98°C, 45 min), immersed in 0.3% H_2_0_2_ and rinsed with 1× PBS before incubation overnight at 4°C with each of the anti-RB1CC1 primary antibodies, rabbit polyclonal antibodies (N1-poly and N3-poly) [3], [10], [12], [17], [18], [19], [20] and mouse monoclonal antibodies (N1-8, N1-216, N3-2, and N3-42). The sections were rinsed with 1× PBS and incubated with the secondary antibody (Simple Stain MAX-PO; Nichirei, Japan) at room temperature for 1 h. The sections were then stained with 3,3′-diaminobenzidine tetrahydrochloride (DAB), and counter-stained with hematoxylin.

### Microscopic evaluation

As previously described [12], [17], [18], [20], the immunohistochemical results for RB1CC1 were first classified into three categories: I, negative staining in both cytoplasm and nuclei; II, positive staining only in cytoplasm; III, positive staining only in nuclei or in nuclei and cytoplasm. Then, RB1CC1 staining grade I–II and III were defined as “negative (−)” and “positive (+),” respectively; i.e., only the cases with nuclear RB1CC1 expression were recognized as RB1CC1(+). Among four kinds of monoclonal antibodies, N1-8 reacted better than the others with formalin-fixed paraffin-embedded tissue sections, so it was applied to compare the clinical utility of a monoclonal antibody with that of an anti-RB1CC1 pre-existing polyclonal antibody [3], [10], [12], [17], [18], [19], [20] in 57 breast cancer cases.
